# Moving to a non-dopaminergic approach for the treatment of OFF fluctuations in Parkinson’s disease

**DOI:** 10.1016/j.prdoa.2025.100303

**Published:** 2025-01-27

**Authors:** Stuart H. Isaacson, Peter Jenner

**Affiliations:** a Parkinson’s Disease and Movement Disorders Center of Boca Raton Boca Raton FL USA; b Institute of Pharmaceutical Sciences, Faculty of Life Sciences and Medicine, King’s College London London UK

**Keywords:** Amantadine, Istradefylline, Motor fluctuations, OFF time

## Abstract

In levodopa treated patients with Parkinson’s disease (PD), the standard approach to managing motor fluctuations is to adjust dopaminergic therapy. However, despite the availability of a wide armamentarium of dopaminergic medications, most patients treated with levodopa will still experience significant OFF time, and it is increasingly clear that motor fluctuations have a significant non-dopaminergic component. In this narrative review, we compare and contrast the therapeutic profiles of the only two non-dopaminergic medications approved in the US for the management of OFF time, namely amantadine and istradefylline. When compared against each other the two agents exemplify two different pharmacological approaches to treatment. Whereas amantadine has a multimodal pharmacology, istradefylline has highly specific actions at A_2A_ receptors which are highly expressed in the indirect pathway of the basal ganglia. We discuss how both offer an important alternative approach to treatment, without increasing total dopaminergic load. Clinicians can also consider that amantadine and istradefylline each have overlapping indications with classic dopaminergic medications, but with distinct mechanisms of action that can complement each other to reduce motor complications in patients already being treated with other dopaminergic agents.

## 1 Introduction

Ever since the discovery that the severe striatal dopamine deficit in Parkinson’s disease (PD) could be rationally treated 4with the dopamine precursor, levodopa (together with a peripheral decarboxylase inhibitor) [1], [2], most antiparkinsonian therapy has been based on either replacing or mimicking the effects of nigrostriatal dopamine. However, the treatment-associated problems of motor fluctuations have been recognized almost equally as long and remain a major problem for many people with PD (PwP). OFF episodes occur when a levodopa dose is not providing symptom benefit and include end-of-dose wearing-off, delayed-ON, no-ON, suboptimal ON, unpredictable ON-OFF, and morning, postprandial, and nocturnal OFF [3]. All of these types of OFF can significantly impact quality of life and disrupt daily activities [4].

Long-acting oral levodopa formulations (e.g. controlled release [CR] levodopa formulations or extended release [ER] carbidopa/levodopa) and non-oral levodopa delivery systems (e.g. intrajejunal infusion) were specifically developed in an attempt to manage motor fluctuations by improving oral levodopa plasma delivery [5]. The longer-acting post-synaptic dopamine agonists (e.g. ER oral formulations of pramipexole and ropinirole, and transdermal rotigotine) were also developed to provide a more continuous stimulation of dopamine receptors [6]. Attempts to reduce the metabolism of central dopamine with selective MAO-B inhibitors (e.g. selegiline, rasagiline, safinamide) and to reduce metabolism of peripheral levodopa with peripheral COMT inhibitors (e.g. entacapone and opicapone) can also improve OFF time. However, despite all of these therapeutic strategies aimed at replenishing striatal dopamine, most PwP treated with levodopa will still experience significant OFF time [7].

## 2 Problems with a dopaminergic-only approach

In levodopa treated patients, the standard approach to treating OFF periods is to adjust dopaminergic therapy to reduce the *duration* of an OFF episode (rather than its severity or the frequency of OFF episodes). This is primarily because of the close temporal association between OFF time and troughs in plasma levodopa levels [8], [9], [10], [11]. According to the classic ‘storage hypothesis’ that underlies current dopaminergic therapeutic strategies [11], the development of OFF fluctuations reflects the progressive loss of presynaptic storage of levodopa and dopamine in striatal dopaminergic neurons. As a consequence, the ability to ‘buffer’ the fluctuations in levodopa concentrations caused by its short half-life (90 min when administered orally with carbidopa) is lost and, consequently, the therapeutic response to levodopa reflects its plasma pharmacokinetics. However, this concept cannot completely explain OFF periods and there is clearly also a post-synaptic component to motor fluctuations as OFF periods also occur following treatment with dopamine agonists [12]. In agreement, recent work in treatment-naïve patients in sub-Saharan Africa suggests that the ‘long-duration’ therapeutic response to levodopa consists of complex mix of both pre-synaptic and post-synaptic mechanisms [13]. As such, we really should be neither surprised nor disappointed that current treatment strategies used to overcome the ‘presynaptic dopamine deficit’ are not enough on their own to abolish OFF periods.

There are at least two clear shortcomings of the perseverating focus on the classical dopaminergic approach to treating OFF episodes. First, most approaches increase overall dopaminergic load, which in turn increases the risk of classical adverse effects, including exacerbation of pre-existing dyskinesia [14]. Neuropsychiatric adverse events (i.e., hallucinations, impulse control disorders, dopamine dysregulation), somnolence, and orthostatic hypotension may also be provoked [14]. The second shortcoming is that while modification of dopaminergic medication reduces OFF time, it seldom eliminates its occurrence [3]. If OFF time is strictly a function of difficult to manage fluctuations in drug concentration, then approaches to delivering therapy in the form of continuous drug delivery providing a constant source of dopaminergic stimulation should effectively address these challenges. While the use of continuous drug delivery methods, such as intra-duodenal levodopa delivery, or most recently, subcutaneous infusions of levodopa or apomorphine has proven to reduce OFF time (to similar extents), *none* were found to completely eliminate OFF even when maximally optimized alone or in combination with oral dopaminergic medications [3], [15], [16], [17], [18]. Thus, it has become increasingly clear that OFF periods are not entirely dopaminergic in nature and must have a significant non-dopaminergic component. It has been suggested that OFF episodes may involve a complex mix of mechanisms and dopaminergic medications may only target one aspect of these [3]. Given the involvement of multiple neurotransmitter pathways within the basal ganglia that are non-dopaminergic in nature, the inability to eliminate motor fluctuations with a dopaminergic approach is again not surprising.

## 3 Non-dopaminergic agents currently available in the US for the treatment of OFF episodes

The reliance on dopamine-centric models of the basal ganglia to explain how PD treatments work ignores the progress made in understanding the complex pathology and biochemistry of PD [19], [20], [21]. It may be of more than passing interest to reconsider therapeutic approaches not in terms of *either–or* but rather to adopt a more comprehensive approach that accepts there are multiple pathways involved in disease progression, and which targets *both* dopaminergic and non-dopaminergic pathways for optimal control of OFF time.

At this point in time, there are two medications licensed in the US for OFF fluctuations with which this approach can be explored, namely amantadine and istradefylline. While preclinical evidence supports the ability of safinamide to block sodium channels *in vitro* thereby affecting glutamatergic function *in vivo* [22], the translation of this potential non-dopaminergic mechanism of action from bench to bedside has never been proven. For this narrative review, we reviewed PubMed and ClinicalTrials.gov for reports of amantadine and istradefylline for the management of motor fluctuations published in English between database inception and July 30, 2024. We used the search terms “amantadine, istradefylline, motor complications, motor fluctuations, and OFF” and reviewed articles reporting clinical trials (of any design, including randomized controlled trials and observational studies).

### 3.1 Amantadine

Amantadine was introduced in 1966 as an antiviral agent for influenza. Its mild antiparkinsonian efficacy was a chance finding when Schwab and colleagues noticed remission of rigidity, tremors, and akinesia in one of their patients when she received amantadine for flu prevention [23]. It was first approved by the FDA for use in PD, parkinsonism, and drug-induced extrapyramidal reactions in 1973, but its efficacy in treating OFF fluctuations was variable. In the 1990′s, its anti-dyskinetic efficacy was explored in smaller trials and its use in clinical practice was not widely adopted due to concerns of dosing, adverse effects, and durability of benefit [24].

Like many serendipitous compounds, amantadine has what has been coined as ‘multimodal’ pharmacology [24]. Its primary use an antidyskinetic was based on the body of preclinical work unpicking the glutamatergic mechanisms underlying levodopa induced dyskinesia (LID) [25], [26] coupled with the work by Kornhuber and colleagues who were systematically evaluating low-affinity NMDA receptor antagonists that could be clinically tolerated [27], [28]. Soon after, work in preclinical models of PD demonstrated amantadine reduced LID [29], and these preclinical findings were rapidly translated into the clinic [30]. However, immediate-release [IR] amantadine was not tested in large Phase III studies to examine its effect on OFF fluctuations. In 2017, a bedtime-administered, combined delayed-release [DR] and ER formulation of amantadine (Gocovri) demonstrated efficacy in reducing dyskinesia and also improved OFF fluctuations [31], [32]. Unlike dopaminergic therapies, this DR/ER formulation is the only drug approved to reduce both LID and OFF episodes [33], [34]. An IR/ER formulation of amantadine (Osmolex) that has similar pharmacokinetics to IR-amantadine did not show efficacy in reducing OFF time [35].

Interestingly, despite being recruited based on troublesome dyskinesia, patients in these phase III trials still had significant OFF time at trial entry approaching 3 h [34]. In these patients, pooled analyses demonstrated approximately 30 % reduction in daily OFF time [34], which is similar to the range of improvement seen with dopaminergic adjuncts such as dopamine agonists, MAO-B and COMT inhibitors [14]. While the durability of amantadine anti-dyskinetic effect has been much debated [36], results from the two year open-label extension of the pivotal trials demonstrated that amantadine-ER-treated participants continued to have maintenance of benefit on both dyskinesia and motor fluctuations for the 2-years trial duration [37]. A recent French network study of patients using IR-amantadine, including those using device aided therapies, demonstrated similar sustained benefit in reducing LID and motor fluctuations over long-term follow-up [38]. Additional improvements in non-motor symptoms, including depression and daytime sleepiness were also noted, although these appeared to track the improvements in motor complications [39]. All of these data should be interpreted with the caveat that the OFF time results are not directly comparable to studies of other adjunct therapies where study participants usually have higher levels (5–6 h) of daily OFF time at baseline [40]. Most patients in the amantadine studies did not have their dopaminergic medications adjusted during the trial period. Table 1 summarizes the recent studies of amantadine which included OFF time as an outcome measure. No prospective studies of amantadine have been performed in the population of PwP typically recruited to OFF fluctuation studies.

**Table 1:** Studies evaluating OFF time reductions with amantadine or istradefylline.

**Study**	**Study design**	**Population**	**Efficacy in reducing OFF time**
**Amantadine**			
EASE LID NCT02136914 [31]	Randomized, double-blind, placebo-controlled clinical trial	Levodopa-treated PD patients with ≥ 1 h of troublesome LID	Significant decrease in OFF time of −0.9 h [95 %CI, −1.6, −0.2] with DR/ER-amantadine 274 mg vs. placebo (p = 0.02)
EASE LID 3 NCT02274766 [32]	Randomized, double-blind, placebo-controlled clinical trial	PD patients with ≥ 1 h of troublesome LID and at least mild functional impact	Significant decrease in OFF time of −1.1 h [95 %CI, −2.0, −0.2] with DR/ER-amantadine 274 mg vs. placebo (p = 0.02)
EASE LID 2 NCT02202551 [37], [67]	2-year open-label trial	Patients who completed the double-blind EASE LID or EASE LID 3 studies	MDS-UPDRS Part IV OFF motor fluctuation subscores remained below double-blind baseline at 1 and 2 years of follow-up.
ALLAY-LID 1 and 2 NCT02153645 NCT02153632 [35]	Randomized, double-blind placebo-controlled trials	PD patients with predictable, problematic and/or disabling peak-effect LID	No significant effect of IR/ER-amantadine on OFF time vs. placebo.
**Istradefylline**			
6002-US-005* NCT00456586 [53]	Randomized, double-blind placebo-controlled trial	PD patients with ≥ 2 h OFF time per day	Significant decrease in percentage of daily OFF time of −6.17 % [95 %CI, −10.60, −1.74] for istradefylline 40 mg/day vs placebo (p = 0.007)
6002-US-006* NCT00456794 [68]			Significant decreases in percentage of daily OFF time of −4.35 % [95 %CI, −8.16, −0.54] (p = 0.026) and −4.49 % [-8.35, −0.62] (p = 0.024) for istradefylline 20 mg/day and 40 mg/day vs placebo, respectively
6002-0608* NCT00455507 [56]			Significant decrease in daily OFF time of −0.65 h [95 %CI, −1.23, −0.07] (p = 0.028) and −0.92 h [95 %CI, −1.49, −0.35] (p = 0.002) for istradefylline 20 mg/day and 40 mg/day vs placebo, respectively.
6002-009* NCT00955526 [55]			Significant decrease in daily OFF time of −076 h [95 %CI, −1.30, −0.22] (p = 0.006) and −0.74 h [95 %CI, −1.27, −0.20] (p = 0.008) for istradefylline 20 mg/day and 40 mg/day vs placebo, respectively.
6002-US-013* NCT00199407 [54]	Randomized, double-blind placebo-controlled trial	PD patients with ≥ 3 h OFF time per day	Significant decrease in percentage of daily OFF time of −4.57 % [95 %CI, −8.55, −0.59] for istradefylline 20 mg/day vs placebo (p = 0.025)
6002-US-018 NCT00199420 [69]			No significant effect on OFF time vs. placebo.
6002-EU-007 NCT00199394 [57]			
6002-014 NCT01968031 [57]	Randomized, double-blind placebo-controlled trial	PD patients with ≥ 2 h OFF time per day and LID	No significant effect on OFF time vs. placebo.

Effect sizes are taken from reference [70].

**Fig. 1:**
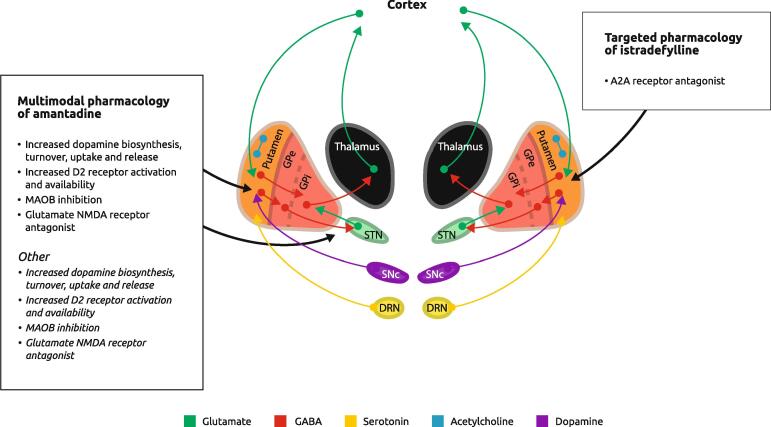
Identified mechanisms of action for amantadine and istradefylline in the treatment of Parkinson’s disease.

Even with its long history of use, the pharmacological mechanisms underlying amantadine’s antiparkinsonian efficacy are not well understood but (in addition to its antiglutamatergic activity) are thought to include several potential dopaminergic and nondopaminergic pathways (Fig. 1) [41]. However, a potential problem for drugs with multiple mechanisms of action is their tolerability profile due to off-target and target activity. Indeed, amantadine has unwanted actions on several central and peripheral neurotransmitter systems. There is a difference between a drug with ‘multimodal’ on target (desired) actions and a drug with off-target, unwanted actions that are associated with adverse events. The main adverse effects of amantadine include hallucinations, orthostatic hypotension, peripheral edema, vivid dreams, sleep fragmentation, falls, dry mouth, and constipation [42]. Livedo reticularis is also a relatively common yet reversible side effect, and amantadine use has also been associated with serious adverse effects, including neuroleptic malignant syndrome, psychosis, suicidal ideation, and depression [42].

With the original IR-formulation, amantadine plasma concentrations are dose-proportional up to 200 mg/day, but higher doses result in disproportional increases in peak and maximum plasma concentrations [43], with a greater likelihood of CNS derived adverse events (e.g. hallucinations and vivid dreams), and other effects which frequently resemble the side effects associated with anticholinergic therapy (e.g. dry mouth and constipation). Indeed, the inconsistency of response and tolerability issues with increasing doses of the classical IR formulation of the drug underpinned the development of the DR/ER formulation designed to provide lower overnight levels with a slower rise in plasma concentrations after a 3–4 h delay, such that maximum concentrations (at plasma levels associated with anti-dyskinesia effect) are achieved upon waking and are sustained throughout the day, while lower concentrations occur in the evening [44]. While the DR/ER formulation of amantadine has improved its tolerability (even at doses comparable to 350 mg of IR amantadine), 20 % of patients discontinued treatment in the trials due to hallucinations or other adverse events. In the amantadine-ER clinical trials hallucinations and falls occurred more frequently in PwP aged 65 years and over, compared to those less than 65 years of age [33]. Renal function must also be considered, especially as the older PD population typically has age related renal impairment which reduces the clearance of amantadine [33].

### 3.2 Istradefylline

The other antiparkinsonian agent with a non-dopaminergic mode of action that is approved in the US (and Japan) is istradefylline (Nourianz), which is approved for use as adjunctive treatment to levodopa/carbidopa in adult patients with PD experiencing OFF episodes [45]. In direct contrast to multimodal amantadine, the mechanism of action of istradefylline is precise and targeted with the drug acting specifically as an adenosine A_2A_ receptor antagonist [46]. It has no known actions on any other neurotransmitter system, or any enzymes associated with the metabolism of levodopa or dopamine. Moreover, A_2A_ receptor expression is limited to the brain – predominantly in the basal ganglia, and even more precisely to the GABAergic spiny projection neurons (SPNs) that project from the striatum to the external globus pallidus [47]. Increases in striatal A_2A_ receptor expression occur early on in the pathogenesis of PD [48] and have been shown to correlate with motor symptoms in PD patients who were previously treated with a wide spectrum of antiparkinsonian drugs [49].

**Fig. 2:**
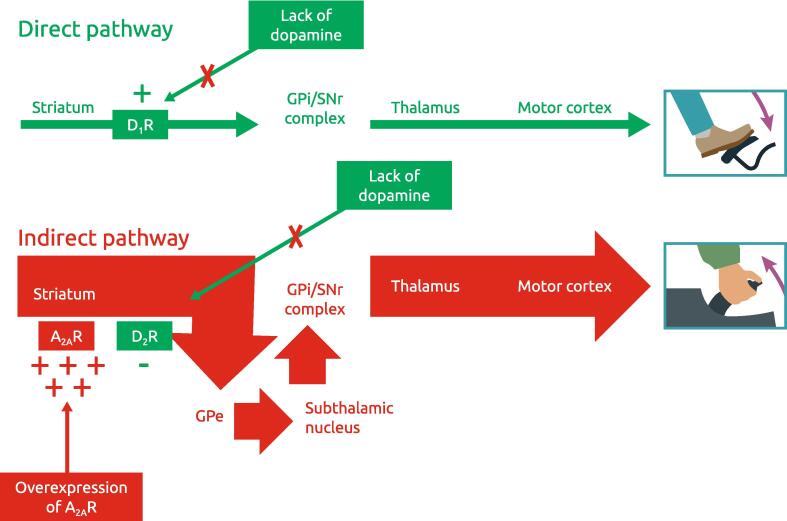
Conceptual representation of the basal ganglia Go (direct) and NO Go (indirect) pathways.

This population of SPNs form part of what is known as the ‘indirect’ or ‘NO GO’ pathway. It is *‘indirect’* because anatomically, the neuronal outflow goes from the striatum to the external pallidum, via the subthalamic nucleus, to the internal pallidum/substantia nigra *pars reticulata*, and then the thalamus. The ‘NO GO’ terminology refers to the function of this basal ganglia pathway in inhibiting movement [50]. At a simplistic level, the indirect pathway works in opposition to the direct pathway (which directly projects from the striatum to the internal pallidum/substantia nigra *pars reticulata)* and acts as the ‘GO’ pathway initiating movement. When both pathways are in balance, there is normal movement [50]. In PwP, dopaminergic degeneration causes the loss of dopaminergic regulation of both basal ganglia pathways. Specifically, the NO GO pathway becomes overactive – thus inhibiting voluntary movement. Dopaminergic drugs act by partially normalizing the dopamine D2 receptor mediated activity of the ‘NO GO’ pathway – but because the A_2A_ system remains overactive (including A_2A_ receptor overexpression [49]), dopamine replacement therapy *cannot* fully normalize the pathway activity. This can be conceptualized by analogy to an automobile – while dopaminergic therapies act to ‘press on the gas pedal’, A_2A_ receptor overactivity acts as ‘having the emergency brake on’ [51], [52] (Fig. 2). In this setting, A_2A_ receptor antagonists effectively ‘release the emergency brake’ of the ‘NO GO’ pathway to facilitate movement. Importantly, A_2A_ receptors are not found on the SPN of the direct ‘GO’ pathway, so should predictably not facilitate dyskinesia.

In the pivotal istradefylline trials leading to US registration, 95 % of participants were already receiving other adjunctive therapy and had improvement in OFF time when istradefylline was added [53], [54], [55], [56]. Within this context, istradefylline reduced OFF time by (a further) 0.75 h for the 20 mg/day dose and 0.82 h for the 40 mg/day dose [57], again in the expected range for adjunct therapies [14]. In parallel, Good ON time (ON time without troublesome dyskinesia) improved by 0.68 h with the 20 mg/day dose and by 0.69 h with the 40 mg/day dose adjusted for placebo [57]. When looking at the effects of istradefylline in PwP *not* already on other adjunct medications, subgroup analyses have shown that study participants receiving istradefylline (20–40 mg/day) as their *first-line* adjunct to levodopa showed a mean placebo-adjusted OFF time reduction of 1.82 h [58] which was larger than the magnitude of effect reported in the overall study population [57]. These data support the recent suggestion that istradefylline may be more effective when given earlier in the disease course [46].

The highly selective and focused pharmacological action of istradefylline likely underlies the demonstrated tolerability and safety profile of istradefylline in the pivotal trials. The only common (>10 %) adverse effect listed in the US prescribing information is dyskinesia (Table 2), which predominantly occurred in patients with pre-existing dyskinesia [57]. Distinct from dopaminergic therapies approved to treat PD OFF fluctuations, istradefylline has not been associated with dopaminergic side effects that often limit dopaminergic therapies. In the pivotal trials, orthostatic hypotension, somnolence, hallucinations, pedal edema, and impulse control disorders was similar for patients receiving istradefylline and placebo. The lower incidence of these troublesome dopaminergic side effects is a clinically important feature that distinguishes istradefylline clinically and can suggest its use in clinical scenarios where PwP have nonmotor symptoms of comorbid conditions, such as orthostatic hypotension, daytime somnolence, cognitive impairment, and/or impulse control disorders [51]. Dyskinesia observed in clinical trials of istradefylline may be due to improved dopaminergic mediated motor function (by ‘releasing the brake’ of the NO GO pathway), but this can usually be controlled by adjusting levodopa levels, especially in PwP with prior dyskinesia. In a pair-wise *meta*-analysis evaluating the risk of dyskinesia and other safety outcomes of other PD medications vs istradefylline, patients taking a dopamine agonist were 1.30 [95 %CI 1.01, 1.69] times more likely to have dyskinesia and 2.50 [95 %CI 1.28, 5.00] times more likely to have somnolence than those taking istradefylline 40 mg/day, and 1.61 [95 %CI 1.16, 2.22] times more likely to have dyskinesia than those taking istradefylline 20 mg/day [59]. Patients taking a COMT inhibitor were 1.52 [95 %CI 1.09, 2.13] times more likely to have dyskinesia than those taking istradefylline 20 mg/day [59].

**Table 2:** Therapeutic profiles of DR/ER amantadine and istradefylline.

	**DR/ER amantadine**	**Istradefylline**
Indication	• Treatment of dyskinesia in PwP receiving levodopa-based therapy, with or without concomitant dopaminergic medications. • Adjunctive treatment to levodopa/carbidopa in PwP experiencing OFF episodes.	Adjunctive treatment to levodopa/carbidopa in adult PwP experiencing OFF episodes
Dose	Initial dose of 137 mg for one week, which is increased to 274 mg	The recommended dosage is 20 mg, which may be increased to a maximum of 40 mg based on individual need and tolerability
Formulation and dosing	DR/ER capsules given at bedtime with an initial delay in (overnight) exposure, followed by sustained amantadine delivery over the dosing interval.	Immediate release tablet given once-daily
Terminal half-life	16 h at steady state	83 h at steady state
Placebo-adjusted OFF episode reduction	• 1 h in pivotal trial population • 1.2 h in patients with > 2.5 h of OFF time at baseline	• 0.75 h for the 20 mg/day dose • 0.82 h for the 40 mg/day dose • 1.82 h as first-line adjunct therapy
Efficacy on non-motor symptoms	In the pivotal studies, MDS-UPDRS Part I daytime sleepiness and depression items were significantly better with amantadine-ER vs placebo.	Pilot studies in PwP and relevant non-motor symptoms at baseline have shown improvements in postural abnormalities, gait disorder, urinary disturbance, and mood disorders.
Common adverse events ≥ 10 %	Hallucinations Dizziness Constipation Peripheral edema Orthostatic hypotension Fall Urinary tract infection	Dyskinesia
Adverse events ≥ 5 %	Anxiety Insomnia Depression/depressed mood Headache Confusion Livedo reticularis Decreased appetite Benign prostatic hyperplasia	Dizziness Constipation Nausea Hallucination Insomnia

In addition to its effects on motor fluctuations, several phase IV studies have examined the effects of istradefylline on non-motor symptoms. The nucleus accumbens (ventral striatum) is considered the neural interface between motivation and action and studies have demonstrated that A_2A_ receptors in this limbic nucleus regulate exertion of effort via the ventral striatopallidal pathway [60]. Several studies show that A_2A_ receptor antagonists can reverse depressive symptoms in experimental models of PD, including models of the motivational symptoms of depression, which may relate to the apathetic/anhedonic expression of depression that occurs in PD [61]. In one small-scale, 12-week study of the effects of istradefylline on mood disorders istradefylline improved symptoms of depression as assessed using the Beck Depression Inventory, the Snaith-Hamilton Pleasure Scale, and the Apathy scale, showing its potential for treating depression in PD [62]. Importantly, and unlike amantadine [39], there was no correlation between the changes in motor function and improvement in depressive symptoms, indicating that the effects on depression are independent of the improvements in motor control [62]. Other small studies have reported benefits of istradefylline in reducing daytime sleepiness [63] and improving freezing of gait [63], [64].

## 4 Clinical implications and future directions: Incorporating non-dopaminergic approaches into the treatment paradigm

While as a strategy, modifying dopaminergic activity in the brain substantially reduces OFF time, it does not fully eliminate it. The use of non-dopaminergic therapies to target glutamatergic (amantadine), adenosergic (istradefylline), and other neurochemical derangements in PD offer an important alternative approach to treatment, without increasing total dopaminergic load and consequent risk of dopamine-related side effects. Clinicians can also consider that amantadine and istradefylline each have overlapping indications with classic dopaminergic medications, but with distinct mechanisms of action that can complement each other to reduce motor complications in patients already being treated with other dopaminergic agents. As we better understand the phenomenology of OFF episodes and their biochemical basis it is likely that a combined pharmacological approach will contribute to addressing the problem of residual OFF time. Until then, non-dopaminergic approaches offer a complementary approach to further reducing what is presumably a non-dopaminergic component of OFF time.

Amantadine and istradefylline can be said to perfectly exemplify two very different pharmacological approaches to treatment (Table 2). Whereas amantadine is known to target NMDA receptors on SPN of both the direct and indirect pathways, as well as affecting multiple neurotransmitters, istradefylline specifically targets A_2A_ receptors largely restricted to the basal ganglia. While the multimodal efficacy of amantadine probably underlies its dual efficacy in both dyskinesia and motor OFF periods [24] (other anti-glutamatergic strategies have been notoriously unsuccessful [24]), it also impacts its safety and tolerability profile. Conversely, the targeted actions of istradefylline are associated with its favorable tolerability profile. Istradefylline also appears to address non-motor symptoms in a manner that is independent of its effects on motor symptoms [62]. Preclinical studies in experimental models of PD indicate that the efficacy of istradefylline is maximal when used with threshold doses of dopaminergic medications [65], [66].

As we enter the era of precision and personalized medicine, all of the currently available treatments for PD will remain important tools in the arsenal of therapies available to clinicians’ treating PwP. Forward-looking towards the emergence of blood and/or neuroimaging biomarkers of distinct non-dopaminergic specific neurochemical derangements will enable precision medicine decision-making in individual PwP. For example, earlier identification of increased adenosine A_2A_ receptor activity in a PwP will suggest istradefylline use. Each pharmacological therapy has a role during the progressive multi-decade course of PD, and treatment paradigms will increasingly reflect a personalized approach and individual response to treatment.

Meanwhile, the recognition that OFF periods occur earlier in the course of the illness than previously thought, worsen with disease progression, involve both motor and non-motor symptoms, persist despite increasing dopaminergic treatment, and are mediated through numerous neurochemical pathways highlights the clinical need for novel non-dopaminergic therapies [3]. Current nonmotor therapies approved for PD have targeted acetylcholine for cognition (rivastigmine), norepinephrine for orthostatic hypotension (droxidopa), and serotonin for PD psychosis (pimavanserin). Perhaps the time has arrived for a shift in the classical dopaminergic treatment paradigm, to an emerging treatment paradigm where treatment strategies extend beyond a singular (dopaminergic) mechanism to instead target multiple distinct mechanisms also involved in the pathophysiology of PD symptoms and motor complications.

## CRediT authorship contribution statement

**Stuart H. Isaacson:** Writing – original draft, Funding acquisition, Conceptualization. **Peter Jenner:** Writing – original draft, Funding acquisition, Conceptualization.

## Declaration of competing interest

The authors declare the following financial interests/personal relationships which may be considered as potential competing interests: Dr. Isaacson reports honoraria for CME, consultant, research grants, and/or promotional speaker on behalf of: Abbvie, Acadia, Acorda, Addex, Affiris, Alexza, Allevion, Amneal, Annovis, Aptinyx, Athira, Bial, Biogen, BlueRock, Britannia, Bukwang, Cala, Cerecor, Cerevel, CND, Eli Lilly, Enterin, Esteve, Fasikl, GE Healthcare, Global Kinetics, Inhibikase, Intra-Cellular Therapies, Ipsen, Jazz, Kyowa Kirin, Lundbeck, Medscape, Merz, Michael J. Fox Foundation, Mitsubishi Tanabe, Neuralys, Neurocrine, NeuroDerm, Neurolive, Exeltis, Novartis, ONO Pharmaceutical, Parkinson Study Group, Pharma2B, Praxis, Revance, Roche, Sage, Sanofi, Scion, Stoparkinson, Sunovion, Sun Pharma, Supernus, Teva, Theravance, Transposon, and UCB. Professor Jenner reports honoraria for consultancy and advisory boards from AbbVie, Bial, Britannia Pharmaceuticals, Cerevel, Kyowa Kirin, and Zambon.

## References

[1] Hornykiewicz O. (2010). A brief history of levodopa. J. Neurol..

[2] Papavasiliou P.S., Cotzias G.C., Düby S.E., Steck A.J., Fehling C., Bell M.A. (1972). Levodopa in Parkinsonism: potentiation of central effects with a peripheral inhibitor. N. Engl. J. Med..

[3] Rota S., Urso D., van Wamelen D.J., Leta V., Boura I., Odin P., Espay A.J., Jenner P., Chaudhuri K.R. (2022). Why do 'OFF' periods still occur during continuous drug delivery in Parkinson's disease?. Transl Neurodegener.

[4] S. Gandhi, T. Zerenner, A. Nodehi, M. Lawton, V. Marshall, F. Al-Hajraf, K. Grosset, H. Morris, M. Hu, Y. Ben-Shlomo, D. Grosset, Motor Complications in Parkinson's Disease: Results from 3343 Patients Followed for up to 12 Years, Movement disorders clinical practice (2024). 10.1002/mdc3.14044 PMC11145112 38587023

[5] LeWitt P.A. (2015). Levodopa therapy for Parkinson's disease: Pharmacokinetics and pharmacodynamics. Mov. Disord..

[6] Olanow C.W., Obeso J.A., Stocchi F. (2006). Drug insight: Continuous dopaminergic stimulation in the treatment of Parkinson's disease. Nat. Clin. Pract. Neurol..

[7] Damier P., Henderson E.J., Romero-Imbroda J., Galimam L., Kronfeld N., Warnecke T. (2022). Impact of Off-Time on Quality of Life in Parkinson's Patients and Their Caregivers: Insights from Social Media. Parkinsons Dis.

[8] Shoulson I., Glaubiger G.A., Chase T.N. (1975). On-off response, Clinical and Biochemical Correlations during Oral and Intravenous Levodopa Administration in Parkinsonian Patients. Neurology.

[9] Stocchi F., Vacca L., Ruggieri S., Olanow C.W. (2005). Intermittent vs continuous levodopa administration in patients with advanced Parkinson disease: a clinical and pharmacokinetic study. Arch. Neurol..

[10] Nyholm D., Lennernäs H., Gomes-Trolin C., Aquilonius S.M. (2002). Levodopa pharmacokinetics and motor performance during activities of daily living in patients with Parkinson's disease on individual drug combinations. Clin. Neuropharmacol..

[11] Kempster P.A., Frankel J.P., Bovingdon M., Webster R., Lees A.J., Stern G.M. (1989). Levodopa peripheral pharmacokinetics and duration of motor response in Parkinson's disease. J. Neurol. Neurosurg. Psychiatry.

[12] Thomas A., Bonanni L., Iorio A.D., Varanese S., Anzellotti F., D’Andreagiovanni A., Stocchi F., Onofrj M. (2006). End-of-dose deterioration in non ergolinic dopamine agonist monotherapy of Parkinson’s disease. J. Neurol..

[13] Cilia R., Cereda E., Akpalu A., Sarfo F.S., Cham M., Laryea R., Obese V., Oppon K., Del Sorbo F., Bonvegna S., Zecchinelli A.L., Pezzoli G. (2020). Natural history of motor symptoms in Parkinson's disease and the long-duration response to levodopa. Brain.

[14] Sisodia V., Dubbeld L., De Bie R.M.A., Duarte G.S., Costa J., Dijk J.M. (2024). Efficacy and safety of adjunctive oral therapy in Parkinson's disease with motor complications: a systematic review and network meta-analysis. BMJ Neurol Open.

[15] Soileau M.J., Aldred J., Budur K., Fisseha N., Fung V.S., Jeong A., Kimber T.E., Klos K., Litvan I., O'Neill D., Robieson W.Z., Spindler M.A., Standaert D.G., Talapala S., Vaou E.O., Zheng H., Facheris M.F., Hauser R.A. (2022). Safety and efficacy of continuous subcutaneous foslevodopa-foscarbidopa in patients with advanced Parkinson's disease: a randomised, double-blind, active-controlled, phase 3 trial. Lancet Neurol..

[16] Espay A.J., Stocchi F., Pahwa R., Albanese A., Ellenbogen A., Ferreira J.J., Giladi N., Gurevich T., Hassin-Baer S., Hernandez-Vara J., Isaacson S.H., Kieburtz K., LeWitt P.A., Lopez-Manzanares L., Olanow C.W., Poewe W., Sarva H., Yardeni T., Adar L., Salin L., Lopes N., Sasson N., Case R., Rascol O., Bou N.S.G. (2024). Safety and efficacy of continuous subcutaneous levodopa-carbidopa infusion (ND0612) for Parkinson's disease with motor fluctuations (BouNDless): a phase 3, randomised, double-blind, double-dummy, multicentre trial. Lancet Neurol..

[17] Olanow C.W., Kieburtz K., Odin P., Espay A.J., Standaert D.G., Fernandez H.H., Vanagunas A., Othman A.A., Widnell K.L., Robieson W.Z., Pritchett Y., Chatamra K., Benesh J., Lenz R.A., Antonini A., Group L.H.S. (2014). Continuous intrajejunal infusion of levodopa-carbidopa intestinal gel for patients with advanced Parkinson's disease: a randomised, controlled, double-blind, double-dummy study. Lancet Neurol..

[18] Katzenschlager R., Poewe W., Rascol O., Trenkwalder C., Deuschl G., Chaudhuri K.R., Henriksen T., van Laar T., Spivey K., Vel S., Staines H., Lees A. (2018). Apomorphine subcutaneous infusion in patients with Parkinson's disease with persistent motor fluctuations (TOLEDO): a multicentre, double-blind, randomised, placebo-controlled trial. Lancet Neurol..

[19] McGregor M.M., Nelson A.B. (2019). Circuit Mechanisms of Parkinson’s Disease. Neuron.

[20] Zhai S., Cui Q., Simmons D.V., Surmeier D.J. (2023). Distributed dopaminergic signaling in the basal ganglia and its relationship to motor disability in Parkinson's disease. Curr. Opin. Neurobiol..

[21] Surmeier D.J., Zhai S., Cui Q., Simmons D.V. (2023). Rethinking the network determinants of motor disability in Parkinson's disease. Front. Synaptic Neurosci..

[22] Sciaccaluga M., Mazzocchetti P., Bastioli G., Ghiglieri V., Cardinale A., Mosci P., Caccia C., Keywood C., Melloni E., Padoani G., Vailati S., Picconi B., Calabresi P., Tozzi A. (2020). Effects of safinamide on the glutamatergic striatal network in experimental Parkinson's disease. Neuropharmacology.

[23] Schwab R.S., England A.C., Poskanzer D.C., Young R.R. (1969). Amantadine in the treatment of Parkinson's disease. J. Am. Med. Assoc..

[24] Rascol O., Fabbri M., Poewe W. (2021). Amantadine in the treatment of Parkinson's disease and other movement disorders. Lancet Neurol..

[25] P. Ravenscroft, J. Brotchie, NMDA receptors in the basal ganglia, J Anat 196 (Pt 4)(Pt 4) (2000) 577-85. 10.1046/j.1469-7580.2000.19640577.x PMC1468098 10923988

[26] Ahmed I., Bose S.K., Pavese N., Ramlackhansingh A., Turkheimer F., Hotton G., Hammers A., Brooks D.J. (2011). Glutamate NMDA receptor dysregulation in Parkinson's disease with dyskinesias. Brain.

[27] Kornhuber J., Bormann J., Hübers M., Rusche K., Riederer P. (1991). Effects of the 1-amino-adamantanes at the MK-801-binding site of the NMDA-receptor-gated ion channel: a human postmortem brain study. Eur. J. Pharmacol..

[28] Kornhuber J., Bormann J., Retz W., Hübers M., Riederer P. (1989). Memantine displaces [3H]MK-801 at therapeutic concentrations in postmortem human frontal cortex. Eur. J. Pharmacol..

[29] Blanchet P.J., Konitsiotis S., Chase T.N. (1998). Amantadine reduces levodopa-induced dyskinesias in parkinsonian monkeys. Mov. Disord..

[30] Luginger E., Wenning G.K., Bösch S., Poewe W. (2000). Beneficial effects of amantadine on L-dopa-induced dyskinesias in Parkinson's disease. Mov. Disord..

[31] Pahwa R., Tanner C.M., Hauser R.A., Isaacson S.H., Nausieda P.A., Truong D.D., Agarwal P., Hull K.L., Lyons K.E., Johnson R., Stempien M.J. (2017). ADS-5102 (Amantadine) Extended-Release Capsules for Levodopa-Induced Dyskinesia in Parkinson Disease (EASE LID Study): A Randomized Clinical Trial. JAMA Neurol..

[32] Oertel W., Eggert K., Pahwa R., Tanner C.M., Hauser R.A., Trenkwalder C., Ehret R., Azulay J.P., Isaacson S., Felt L., Stempien M.J. (2017). Randomized, placebo-controlled trial of ADS-5102 (amantadine) extended-release capsules for levodopa-induced dyskinesia in Parkinson's disease (EASE LID 3). Mov. Disord..

[33] GOCOVRI (amantadine) extended release capsules, for oral use. Available at https://www.accessdata.fda.gov/drugsatfda_docs/label/2017/208944lbl.pdf.

[34] Elmer L.W., Juncos J.L., Singer C., Truong D.D., Criswell S.R., Parashos S., Felt L., Johnson R., Patni R. (2018). Pooled Analyses of Phase III Studies of ADS-5102 (Amantadine) Extended-Release Capsules for Dyskinesia in Parkinson's Disease. CNS Drugs.

[35] Rascol O., Tonges L., deVries T., Jaros M., Quartel A., Jacobs D., Allay-Lid I., Groups A.-L.I.s. (2022). Immediate-release/extended-release amantadine (OS320) to treat Parkinson's disease with levodopa-induced dyskinesia: Analysis of the randomized, controlled ALLAY-LID studies. Parkinsonism Relat. Disord..

[36] Thomas A., Iacono D., Luciano A.L., Armellino K., Di Iorio A., Onofrj M. (2004). Duration of amantadine benefit on dyskinesia of severe Parkinson's disease. J. Neurol. Neurosurg. Psychiatry.

[37] Tanner C.M., Pahwa R., Hauser R.A., Oertel W.H., Isaacson S.H., Jankovic J., Johnson R., Chernick D., Hubble J. (2020). EASE LID 2: A 2-Year Open-Label Trial of Gocovri (Amantadine) Extended Release for Dyskinesia in Parkinson's Disease. J. Parkinsons Dis..

[38] Fabbri M., Rousseau V., Corvol J.-C., Sommet A., Tubach F., De Rycke Y., Bertille N., Selvarasa Y., Carvalho S., Chaigneau V., Brefel-Courbon C., Ory-Magne F., Tessier S., Tir M., Bereau M., Meissner W.G., Thiriez C., Marques A., Remy P., Schneider V., Moro E., Defebvre L., Houeto J.L., Prange S., Eusebio A., Geny C., Frismand S., Damier P., Reuther C.G., Castelnovo G., Benatru I., De Maindreville A.D., Drapier S., Maltête D., Lagha-Boukbiza O., Rascol O., Aubignat M., Magnin E., Burbaud P.P., Guehl P.D., Foubert-Samier A., Laurens B., Boraud T., Vergnet S., Bendetowicz D., Palpacuer T., Debilly B., Derost P., Beal C., Salhi H., Dormeuil A., Petit A., Gravier A., Dupont G., Garnier L., Fraix V., Castrioto A., Meoni S., Carriere N., Danaila T., Laurencin C., Thobois S., Azulay J.-P., Fluchere F., Charif M., Picot M.-C., Hopes L., Corbille A.-G., Rouaud T., Derkinderen P., Alecu C., Heraud C., De Verdal M., Degos B., Mangone G., Sambin S., Lanore A., Courtin T., Mariani L.-L., Bendetowicz D., Khoury F., Menon P., Cormier-Dequaire F., Flamand-Roze E., Grabli D., Hainque E., Vidhaillet M., Meneret A., Delorme C., Foucard C., Von Raison F., Elbaz A., Hartmann A., Leclercq V., Ansquer S., Leh F., Leclercq M., Costentin G., Lagha B., Brefel Courbon C., Leung C., Catala H., Causel A., Gaiffe E., Dupouy S., Villars S., Lai W.-H., Bari R., Chevanne D., Durand E., Rieu I., Bernard S., Garsault C., Boudjema N., Grebent P., Kistner A., Pelissier P., Santraine V., Gaudin T., Boutet P., Caire C., Nouira M., Verna C., Jardel A., Puisieux S., Clement G., Le Monnier L., Frenais R., Le Dily S., Chaigneau R., Ferrier V., David E., Fra L., Foucaran E., Dongmo-Kenfack C., Beauzor F., Le M., Messar S., Liot S., Rabois E., Bonnaire-Verdier M., Kestens F., Gourhan R., Lopez-Alfaro S., Houvenaghel J.-F., Alexandre M., Bourdonnais C., Vernon L., Boumediene A., Julie C., Lobstein A., Longato N., Mitterle M.-P., Philips C., Rummel H., Bras S., Harroch E., Gillet C. (2024). N.S.P.n. for the French, Amantadine use in the French prospective NS-Park cohort. J. Neural Transm..

[39] Mehta S.H., Pahwa R., Tanner C.M., Hauser R.A., Johnson R. (2021). Effects of Gocovri (Amantadine) Extended Release Capsules on Non-Motor Symptoms in Patients with Parkinson's Disease and Dyskinesia. Neurol. Ther..

[40] Isaacson S.H., Pagan F.L., Lew M.F., Pahwa R. (2022). Should “on-demand” treatments for Parkinson's disease OFF episodes be used earlier?. Clin Park Relat Disord.

[41] Danysz W., Dekundy A., Scheschonka A., Riederer P. (2021). Amantadine: reappraisal of the timeless diamond-target updates and novel therapeutic potentials. J Neural Transm (vienna).

[42] Chang C., Ramphul K. (2024).

[43] Symmetrel (Amantadine Hydrochloride) Syrup and Tablets. Available at https://www.accessdata.fda.gov/drugsatfda_docs/label/2009/016023s041,018101s016lbl.pdf.

[44] Hauser R.A., Pahwa R., Wargin W.A., Souza-Prien C.J., McClure N., Johnson R., Nguyen J.T., Patni R., Went G.T. (2019). Pharmacokinetics of ADS-5102 (Amantadine) Extended Release Capsules Administered Once Daily at Bedtime for the Treatment of Dyskinesia. Clin. Pharmacokinet..

[45] NOURIANZ™ (istradefylline) tablets, for oral use. Prescribing information available at https://www.accessdata.fda.gov/drugsatfda_docs/label/2019/022075s000lbl.pdf.

[46] Jenner P., Mori A., Aradi S.D., Hauser R.A. (2021). Istradefylline - a first generation adenosine A2A antagonist for the treatment of Parkinson's disease. Expert Rev. Neurother..

[47] Rosin D.L., Hettinger B.D., Lee A., Linden J. (2003). Anatomy of adenosine A2A receptors in brain: morphological substrates for integration of striatal function. Neurology.

[48] Villar-Menéndez I., Porta S., Buira S.P., Pereira-Veiga T., Díaz-Sánchez S., Albasanz J.L., Ferrer I., Martín M., Barrachina M. (2014). Increased striatal adenosine A2A receptor levels is an early event in Parkinson’s disease-related pathology and it is potentially regulated by miR-34b. Neurobiol. Dis..

[49] Varani K., Vincenzi F., Tosi A., Gessi S., Casetta I., Granieri G., Fazio P., Leung E., MacLennan S., Granieri E., Borea P.A. (2010). A2A adenosine receptor overexpression and functionality, as well as TNF-alpha levels, correlate with motor symptoms in Parkinson's disease. FASEB J..

[50] Mori A. (2020). How do adenosine A<sub>2A</sub> receptors regulate motor function?. Parkinsonism Relat. Disord..

[51] Isaacson S.H., Betté S., Pahwa R. (2022). Istradefylline for OFF Episodes in Parkinson's Disease: A US Perspective of Common Clinical Scenarios. Degener Neurol Neuromuscul Dis.

[52] Mori A., Chen J.F., Uchida S., Durlach C., King S.M., Jenner P. (2022). The Pharmacological Potential of Adenosine A(2A) Receptor Antagonists for Treating Parkinson's Disease. Molecules.

[53] LeWitt P.A., Guttman M., Tetrud J.W., Tuite P.J., Mori A., Chaikin P., Sussman N.M., Group U.S.S. (2008). Adenosine A2A receptor antagonist istradefylline (KW-6002) reduces “off” time in Parkinson's disease: a double-blind, randomized, multicenter clinical trial (6002-US-005). Ann. Neurol..

[54] Hauser R.A., Shulman L.M., Trugman J.M., Roberts J.W., Mori A., Ballerini R., Sussman N.M., Istradefylline U.S.S.G. (2008). Study of istradefylline in patients with Parkinson's disease on levodopa with motor fluctuations. Mov. Disord..

[55] Mizuno Y., Kondo T., Japanese Istradefylline Study G. (2013). Adenosine A2A receptor antagonist istradefylline reduces daily OFF time in Parkinson's disease. Mov. Disord..

[56] Mizuno Y., Hasegawa K., Kondo T., Kuno S., Yamamoto M., Japanese Istradefylline Study G. (2010). Clinical efficacy of istradefylline (KW-6002) in Parkinson's disease: a randomized, controlled study. Mov. Disord..

[57] Hauser R.A., Hattori N., Fernandez H., Isaacson S.H., Mochizuki H., Rascol O., Stocchi F., Li J., Mori A., Nakajima Y., Ristuccia R., LeWitt P. (2021). Efficacy of Istradefylline, an Adenosine A2A Receptor Antagonist, as Adjunctive Therapy to Levodopa in Parkinson's Disease: A Pooled Analysis of 8 Phase 2b/3 Trials. J. Parkinsons Dis..

[58] P. LeWitt, N. Hattori, A. Mori, K. Toyama, E. Ohta, P. Salzman, S.H. Isaacson, Efficacy of Istradefylline, an A2A Receptor Antagonist, When Added to Levodopa (LD) and Other Medications for Parkinson’s Disease (PD) [abstract]. Mov Disord. 2019; 34 (suppl 2). https://www.mdsabstracts.org/abstract/efficacy-of-istradefylline-an-a2a-receptor-antagonist-when-added-to-levodopa-ld-and-other-medications-for-parkinsons-disease-pd/. Accessed July 30, 2024.

[59] Torres-Yaghi Y., Qian J., Cummings H., Shimoda H., Ito S., Batson S., Mitchell S., Pagan F. (2024). Comparative Safety of Istradefylline Among Parkinson’s Disease Adjunctive Therapies: A Systematic Review and Meta-analysis of Randomized Controlled Studies (P5-3.017). Neurology.

[60] Mingote S., Font L., Farrar A.M., Vontell R., Worden L.T., Stopper C.M., Port R.G., Sink K.S., Bunce J.G., Chrobak J.J., Salamone J.D. (2008). Nucleus accumbens adenosine A2A receptors regulate exertion of effort by acting on the ventral striatopallidal pathway. J. Neurosci..

[61] Jenner P., Mori A., Kanda T. (2020). Can adenosine A(2A) receptor antagonists be used to treat cognitive impairment, depression or excessive sleepiness in Parkinson's disease?. Parkinsonism Relat. Disord..

[62] Nagayama H., Kano O., Murakami H., Ono K., Hamada M., Toda T., Sengoku R., Shimo Y., Hattori N. (2019). Effect of istradefylline on mood disorders in Parkinson's disease. J. Neurol. Sci..

[63] Matsuura K., Kajikawa H., Tabei K.I., Satoh M., Kida H., Nakamura N., Tomimoto H. (2018). The effectiveness of istradefylline for the treatment of gait deficits and sleepiness in patients with Parkinson's disease. Neurosci. Lett..

[64] Iijima M., Orimo S., Terashi H., Suzuki M., Hayashi A., Shimura H., Mitoma H., Kitagawa K., Okuma Y. (2019). Efficacy of istradefylline for gait disorders with freezing of gait in Parkinson's disease: A single-arm, open-label, prospective, multicenter study. Expert Opin. Pharmacother..

[65] Uchida S., Tashiro T., Kawai-Uchida M., Mori A., Jenner P., Kanda T. (2014). Adenosine A_2_A-receptor antagonist istradefylline enhances the motor response of L-DOPA without worsening dyskinesia in MPTP-treated common marmosets. J. Pharmacol. Sci..

[66] Uchida S., Soshiroda K., Okita E., Kawai-Uchida M., Mori A., Jenner P., Kanda T. (2015). The adenosine A2A receptor antagonist, istradefylline enhances anti-parkinsonian activity induced by combined treatment with low doses of L-DOPA and dopamine agonists in MPTP-treated common marmosets. Eur. J. Pharmacol..

[67] Hauser R.A., Lytle J., Formella A.E., Tanner C.M. (2022). Amantadine delayed release/extended release capsules significantly reduce OFF time in Parkinson's disease. NPJ Parkinsons Dis.

[68] Stacy M., Silver D., Mendis T., Sutton J., Mori A., Chaikin P., Sussman N.M. (2008). A 12-week, placebo-controlled study (6002-US-006) of istradefylline in Parkinson disease. Neurology.

[69] Pourcher E., Fernandez H.H., Stacy M., Mori A., Ballerini R., Chaikin P. (2012). Istradefylline for Parkinson's disease patients experiencing motor fluctuations: results of the KW-6002-US-018 study. Parkinsonism Relat. Disord..

[70] Center for drug evaluation and research. Clinical Review: NDA 022075. Available at https://www.accessdata.fda.gov/drugsatfda_docs/nda/2019/022075Orig1s000MedR.pdf Last accessed January 9, 2025.

